# Architect of Frailty Biology and Champion of Translational Geroscience

**DOI:** 10.1111/acel.70406

**Published:** 2026-02-10

**Authors:** Peter M. Abadir, Brian Buta, Karen Bandeen‐Roche

**Affiliations:** ^1^ Department of Medicine, Division of Geriatric Medicine and Gerontology Johns Hopkins University Baltimore Maryland USA; ^2^ Department of Biostatistics, Johns Hopkins Bloomberg School of Public Health Johns Hopkins University Baltimore Maryland USA

## Abstract

Jeremy Walston helped transform frailty from a clinical syndrome into a biologically grounded condition, linking chronic inflammation, mitochondrial dysfunction, and impaired stress responses to loss of physiologic reserve. His leadership of the Johns Hopkins OAIC and SPRING studies advanced translational geroscience and trained a generation of investigators.

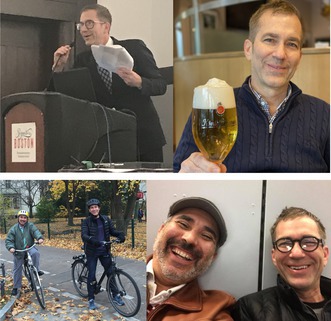

## 1 Jeremy Walston, MD (1959–2025): Architect of Frailty Biology and Geroscience Bridge‐Builder

Dr. Jeremy Walston, a towering figure in aging research, passed away in June 2025. His legacy lies not only in the scientific frameworks he helped establish, but in the durable institutions, conceptual models, and community of investigators he shaped over three decades. Dr. Walston was a pioneer in defining the biological underpinnings of frailty, introducing the field to translational aging biology long before it became mainstream (Fountain et al. 2024; Perazza et al. 2022; Ijaz et al. 2022; Dent et al. 2019; Morley et al. 2013; Walston, Hadley, et al. 2006). As Principal Investigator of the Johns Hopkins Older Americans Independence Center (OAIC) for over 17 years, he elevated frailty from a clinical observation to a biological construct, seeding new domains of inquiry at the intersection of inflammation, mitochondrial energetics, resilience, and geroscience (Perazza et al. 2022; Zampino et al. 2020; Sierra et al. 2021; Ferrucci et al. 2024; Kim and Rockwood 2024). This tribute reflects on the historical arc of Dr. Walston's work and the impact of his vision.

## 2 The Emergence of Frailty Biology: The Role of Chronic Inflammation

Frailty, once described vaguely as “failure to thrive” in old age, lacked a clear biological identity until the late 1990s. Working in parallel with Dr. Linda Fried and others, Dr. Walston contributed to the empirical and conceptual foundations of the frailty phenotype, defined by shrinking, weakness, slowness, exhaustion, and low activity (Walston, Hadley, et al. 2006; Fried et al. 2001, 2021). Yet his contributions extended beyond phenotype construction: he asked what these features meant biologically. Why do certain older adults lack physiological reserve and show exaggerated vulnerability to stressors?

This question became the through line of his scientific life: How does biological aging confer vulnerability? (Morley et al. 2013; Fried et al. 2021; Walston et al. 2018; Dent et al. 2019; Phillip et al. 2015).

Dr. Walston's early research offered one of the first major biological signatures of frailty, demonstrating the elevation of pro‐inflammatory cytokines, particularly interleukin‐6 (IL‐6), in frail older adults (Leng et al. 2002). His 2007 Journal of American Geriatrics Society article “Inflammation and Frailty in Older Women,” (Leng et al. 2007) was a turning point. It provided molecular evidence anchoring to the phenotype and ushered in a new era in which frailty could be quantified biologically, not just clinically. Subsequent work by his group extended this finding to tumor necrosis factor‐alpha (TNF‐α), C‐reactive protein (CRP), and other components of the chronic inflammation cascade (Westbrook et al. 2020; Nidadavolu et al. 2022, 2025, 2023).

**FIGURE 1:**
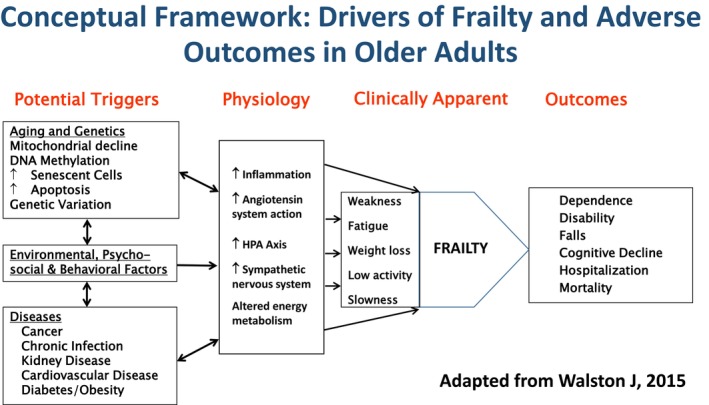
Conceptual Framework developed by Dr. Walston: Biological, Environmental, and Physiologic Drivers of Frailty and Adverse Outcomes in Older Adults. This framework illustrates the multiscale determinants of frailty as articulated and advanced by Dr. Jeremy Walston. Key upstream influences—including aging biology, genetic variation, mitochondrial dysfunction, DNA methylation, cellular senescence, apoptosis, and chronic inflammation—interact with environmental, psychosocial, and behavioral factors to shape physiologic vulnerability. Core dysregulated pathways central to frailty physiology (e.g., inflammation, angiotensin system activity, HPA axis activation, sympathetic nervous system signaling, and impaired energy metabolism) give rise to the clinical frailty phenotype characterized by weakness, fatigue, weight loss, low activity, and slowness. These processes collectively increase risk for dependence, disability, falls, cognitive decline, hospitalization, and mortality.

Dr. Walston framed these inflammatory changes not simply as correlates of frailty, but as contributors to its pathophysiology. His hypothesis that chronic low‐grade inflammation erodes physiological reserve influenced a generation of aging research and opened the door to studies of immune senescence, metabolic dysregulation, and multimorbidity as interlocking features of frailty (Figure 1) (Fried et al. 2001; Walston et al. 2002).

As Dr. Walston's career progressed, he saw that mechanistic data were essential in order to gain actionable insights to addressing inflammation in frailty. He proceeded to develop a frail mouse model that does not express the anti‐inflammatory interleukin‐10 molecule (J Gerontol A Biol Sci Med Sci 2008 Apr; 63 (4): 391‐8), and then to characterize it in subsequent papers. Many important findings have followed from this development—and related models—in Dr. Walston's lab, which include identifying kynurenine as a major mediator of neuromuscular dysfunction associated with chronic inflammation and aging and investigating the promise of Losartan treatment to address frailty (Westbrook et al. 2020; Lee et al. 2022).

## 3 Diving Deeper: Frailty Biology and Cellular Energetics

Dr. Walston elucidated roles of cellular energetics as yet another, towering contribution to frailty etiology. His work on mitochondrial dysfunction in frailty, particularly in immune cells and skeletal muscle, helped bridge the gap between geroscience and functional decline. He was among the first to describe mitochondrial bioenergetic impairments in monocytes of frail older adults, linking energetic inefficiency to systemic inflammation and sarcopenia (Howard et al. 2007; Semba et al. 2007a, 2007b; Espinoza et al. 2008; Abadir et al. 2011).

His group applied high‐resolution respirometry, transcriptomics, and proteomics to characterize frailty‐associated changes in mitochondrial structure and function. In doing so, Dr. Walston provided mechanistic insight into how impaired ATP generation, altered redox status, and defective mitophagy might drive the phenotypic features of frailty (Ashar et al. 2015; Burks et al. 2015; Nidadavolu et al. 2021). Translational work his lab led developed a mitochondria‐targeted delivery system to effectively transport angiotensin type‐1 receptor blocker‐Losartan (mtLOS) into the inner mitochondrial membrane and elucidated associations of cell‐free circulating mitochondrial DNA with frailty and both physical and cognitive dysfunction (Nidadavolu et al. 2022, 2025; Phillip et al. 2022).

## 4 The JHU OAIC as a Translational Engine/Powerhouse

As Principal Investigator of the Johns Hopkins OAIC (funded continuously from 2003 to present), Dr. Walston transformed the center into a national hub for translational aging biology. He championed a team‐science approach, recruiting experts in molecular biology, biostatistics, engineering, and geriatrics to study frailty from multiple angles. In partnership with Dr. Karen Bandeen‐Roche, the center developed one of the most sophisticated frailty phenotyping infrastructures in the country, combining clinical assessments, molecular measures, and biosignal acquisition (Ashar et al. 2015; Artz et al. 2014; Langdon et al. 2014; Lin et al. 2014; Ellis et al. 2014; Brown et al. 2014, 2015; Lin et al. 2015; McAdams‐DeMarco et al. 2015; Interleukin 1 Genetics Consortium 2015).

He also established novel cores within the OAIC, including a Biological Mechanisms Core that harmonized omics, biosignals, and inflammation markers across cohorts. His vision of frailty was resolutely multiscale from cytokines to clinical trajectories, and he insisted on linking mechanistic findings to meaningful functional outcomes.

Dr. Walston's OAIC also became a launchpad for geroscience. He forged links with the National Institute on Aging's Geroscience Interest Group and positioned the Hopkins OAIC as a foundational site for testing biologically informed interventions. His leadership shaped the broader OAIC network, where he served as a national leader, mentor, and scientific sounding board for new center investigators.

## 5 Mentorship and Field‐Building

Beyond his publications and grants, Dr. Walston's most lasting impact may lie in his mentorship. He trained over 100 mentees across geriatrics, gerontology, epidemiology, and molecular biology. Many now lead their own research programs and centers, often carrying forward the themes of frailty, resilience, and biological aging. He was an active mentor in the National Institute on Aging's GEMSSTAR, Beeson, and Butler‐Williams programs, and his support for young investigators was unfailing.

His mentoring style combined scientific rigor with quiet encouragement. He asked incisive questions but never domineered. He gave trainees space to find their own voice while offering an unshakable grounding in biological plausibility, careful experimental design, and translational relevance.

Dr. Walston also built field‐wide infrastructure. He co‐led the Biology of Healthy Aging Working Group at Johns Hopkins, supported the Biology Core of the Nathan Shock Center, and co‐developed frailty assessment instruments still in use today. He was co‐author on several key consensus documents, including NIH white papers on frailty and resilience (Walston et al. 2023; Varadhan et al. 2018).

## 6 Physical Resilience, and Multi‐Omics Approaches

In the last decade of his life, Dr. Walston pursued two seminal advancements: elaborating the stress‐response dysregulation hypothesis to encompass resilience as well as frailty, and integrating multi‐omics data (transcriptomic, proteomic, metabolomic) with frailty and resilience trajectories. Working together with key collaborators, he developed frameworks to address the hypothesis that frailty is rooted broadly in dysregulated stress response (Leng, Cappola, et al. 2004; Leng, Yang, and Walston 2004; Semba et al. 2005; Schmaltz et al. 2005; Leng, Xue, Huang, Semba, et al. 2005; Walston et al. 2005; Reiner et al. 2005; Leng, Xue, Huang, Ferrucci, et al. 2005). He investigated stress‐activated pathways such as p38 MAPK and NF‐κB and elucidated how these responses were blunted or dysregulated in frail individuals. Dr. Walston argued that aging and frailty are best understood as a loss of adaptive homeostasis (Walston, Hadley, et al. 2006; Fried et al. 2005; Walston, Xue, et al. 2006; Ray et al. 2006; Bandeen‐Roche et al. 2006; Ble et al. 2006; Arking et al. 2006; Cappola et al. 2006; Lange et al. 2006; Fitzpatrick et al. 2007; Yao et al. 2011).

He led the creation of the Study of Physical Resilience and Aging (SPRING), designed to understand how older adults respond, or fail to respond, to acute physiologic challenges. This study developed experimental paradigms to study acute stressors, implementing hypotheses that the frailty‐resilience spectrum is rooted in physiological system dynamics and dynamic stress testing may reveal more about underlying physiology than static biomarkers alone. He developed a resilience framework that examined how older adults recover from acute stressors (e.g., hospitalization, surgery), identifying predictors of recovery and functional decline. SPRING combined blood‐based biomarkers with wearable sensor data, sleep metrics, and physical activity profiles to quantify resilience phenotypes in real‐world settings (Walston et al. 2023; Varadhan et al. 2018).

Dr. Walston collaborated closely with engineers, informaticians, and biostatisticians to develop predictive models linking molecular signatures to frailty progression as well as resilience. His emphasis on physiological integration—how inflammation, energetics, hormonal signaling, and cognition interrelate—remains a blueprint for current geroscience efforts. He also supported experimental therapeutics, including GLP‐1 receptor agonists, anti‐inflammatory monoclonal antibodies, and mitochondrial enhancers, often piloted in the Hopkins OAIC. His lab's longitudinal cohort work, the Healthy Aging Studies unit, with deep phenotyping of older adults before and after stressors, set a benchmark for translational trial design in aging.

Dr. Jeremy Walston's career represents a scientific arc that transformed frailty from a descriptive syndrome to a biologically grounded condition. He taught the field to look beneath the surface of physical decline and search for upstream regulators, adaptive failures, and inter‐system signaling breakdowns.

His frameworks for biological reserve, physiologic dysregulation, and resilience continue to guide clinical trials, basic aging research, and health system models for older adults. His mentorship created a lineage of investigators whose work spans omics, digital health, immunosenescence, and clinical geriatrics. And his insistence on linking biology to function ensures that his work continues to inform how we treat aging bodies and minds in clinical practice.

As we look ahead, the baton passes to a new generation of scientists. Dr. Walston's example invites us to ask deeper questions, build cross‐disciplinary bridges, and center the older adult in every research endeavor. His work reminds us that the biology of aging is not just molecular; it is human, dynamic, and recoverable.

We honor Dr. Jeremy Walston not only by remembering his achievements, but by continuing his mission: to understand aging not as decline, but as adaptation, and to build a science of resilience worthy of the lives it seeks to serve.

## Author Contributions

All authors contributed equally to the conception, drafting, and critical revision of this manuscript and approved the final version for submission.

## Funding

The authors have nothing to report.

## Conflicts of Interest

The authors were each professionally and personally influenced by Dr. Jeremy Walston as colleagues, mentees, and friends, and write this article in tribute to his scientific and mentoring legacy. Other than these relationships, the authors declare no conflicts of interest.

## Data Availability

The authors have nothing to report.
